# Large Mucinous Borderline Ovarian Tumor in an Asymptomatic Woman: A Case Report

**DOI:** 10.7759/cureus.91805

**Published:** 2025-09-07

**Authors:** Maria Troupi, Spyridon Koliopoulos, Amir Shihada, Alexandros Samolis, Dimosthenis Chrysikos, Theodore Troupis

**Affiliations:** 1 Department of Anatomy, National and Kapodistrian University of Athens, Athens, GRC; 2 Department of Surgery, University of Athens Medical School, Athens, GRC

**Keywords:** atypical proliferative tumor, borderline ovarian tumors, large ovarian tumor, low malignant potential tumor, ovarian mucinous carcinoma, ovarian tumors

## Abstract

Borderline ovarian tumors represent a unique subset of ovarian tumors. Compared to malignant ovarian cancers, borderline ovarian tumors typically affect a younger patient group and have growth patterns and cytological features that fall between benign and malignant tumors. Because there aren't any clinical signs in the early stages, these tumors are usually discovered by chance during a physical examination. There are very few reports of large mucinous borderline ovarian tumors in the literature, and they present different clinical manifestations. Herein, we report the case of a 61-year-old asymptomatic woman with a large cystic mass initially detected by ultrasonography. Magnetic resonance imaging and computed tomography examinations showed a large lesion with a maximum diameter of 24 cm, which stemmed from the right ovary and occupied the entire abdomen. Of the tumor markers analyzed, carcinogen antigen 125 levels were found to be elevated. The patient underwent exploratory laparotomy, and the tumor was removed en bloc. Total hysterectomy with bilateral salpingo-oophorectomy and appendectomy was also performed. The pathological examination revealed a cystic neoplasm measuring 25 x 18 x 16 cm and weighing 4 kg, and the histologic study established the diagnosis of a mucinous ovarian neoplasm of borderline malignancy. Despite the size of the tumor, the case described here shows that the patient had a good prognosis because there were no symptoms at diagnosis for a borderline mucinous histotype. Large ovarian lesions are often related to compressive symptoms and require resection soon after diagnosis to ensure a good prognosis.

## Introduction

Borderline ovarian tumors are considered a distinct clinical entity defined histologically by atypical epithelial proliferation without stromal invasion, which differentiates them from ovarian carcinoma [1]. Borderline ovarian tumors represent 10-20% of all ovarian epithelial tumors, are usually found in young patients, and they often have a better prognosis and a less severe clinical course than malignant tumors [1,2]. The majority of these tumors are histologically serous or mucinous type [1,2]. Clinical signs and symptoms, such as stomach discomfort and growing abdominal distention, are frequently ambiguous and appear late in the course of the illness. Many patients have no obvious clinical manifestations, and preoperative diagnosis can be challenging because clinical and ultrasonographic features might overlap with invasive carcinomas [1-3]. An assessment of clinical presentation, physical examination, radiological, and biochemical findings is necessary to tailor management strategies for patients with ovarian tumors.

Here, we present the case of a 61-year-old woman with a large mucinous borderline ovarian tumor. The absence of clinical manifestations, the advanced age of the patient, which is uncommon for this lesion type, and the rarity of such large lesions reported in the literature constitute the significance of this case presentation.

## Case presentation

This is the case of a 61-year-old woman who presented with weight gain and a gradual increase in the abdominal girth within the last year. In a routine ultrasonography of the lower abdomen, a large amount of fluid in the abdomen (ascites) was initially observed, while computed tomography depicted a large cystic lesion, which had occupied the entire abdomen. Biopsy of the lesion did not confirm any disease, and the patient was admitted to the hospital for further examination.

**Figure 1:**
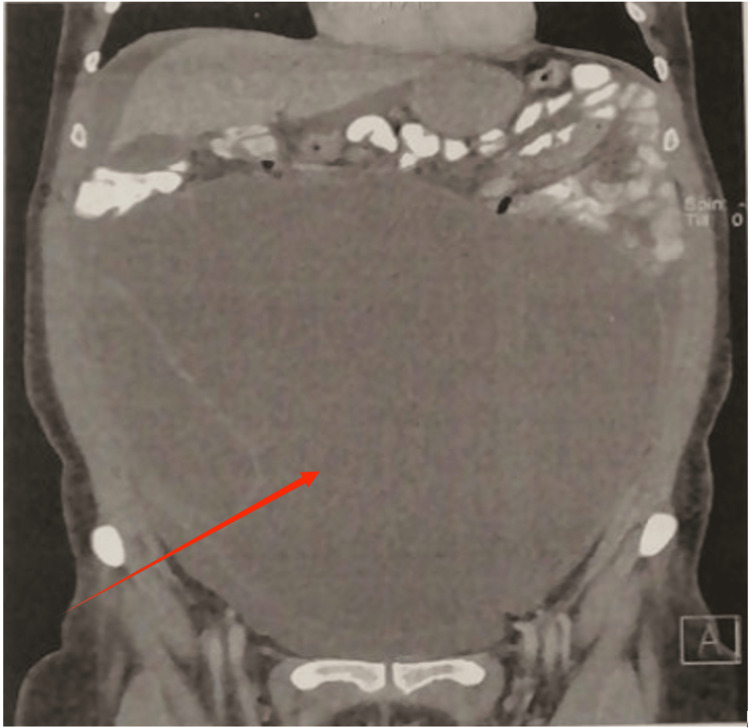
Coronal abdominal magnetic resonance imaging (MRI) revealed a large, near-water-density cystic mass with internal thick septations and mural calcification (red arrow).

**Figure 2:**
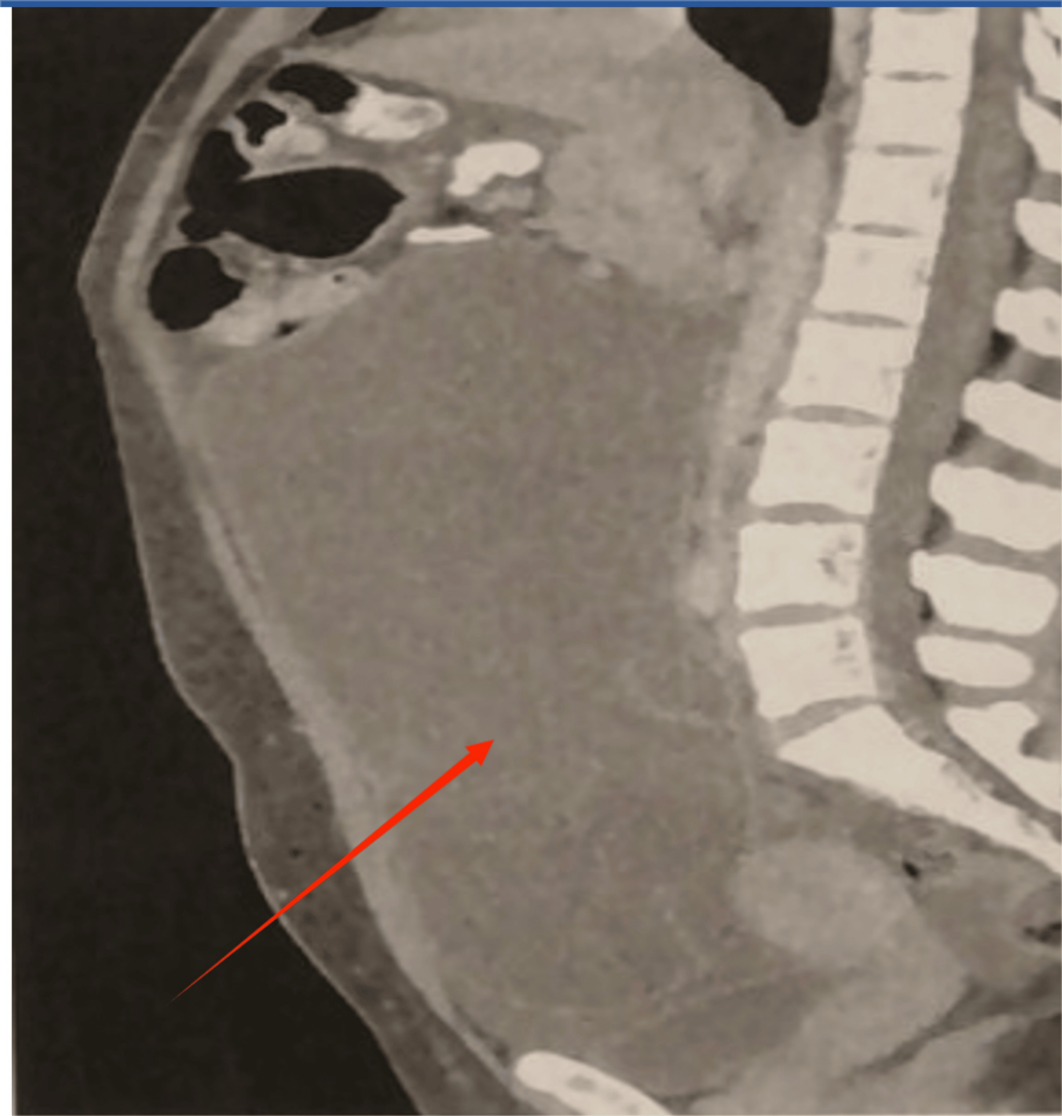
Sagittal abdominal magnetic resonance imaging (MRI) revealed a large, near-water-density cystic mass with internal thick septations and mural calcification (red arrow).

Abdominal magnetic resonance imaging revealed a large, near-water-density fluid-filled cystic mass with internal thick septa and mural calcification. The lesion, with a maximum diameter of 24 cm, stemmed from the right ovary and extended from the pelvis up to the upper abdomen. The endometrium was depicted as thickened and heterogeneous. Other organs, including the kidneys, gall bladder, and liver, were unremarkable. No nodal involvement or peritoneal implants were identified. There were no significant illnesses or cancers mentioned in her personal or family background. Pseudomyxoma peritonei and ovarian cancer were among the differential diagnoses made by radiology and clinical evaluation (Figures 1, 2). Blood tests are presented in Table 1.

**Table 1:** Laboratory tests.

Blood test	Values	Reference range
Hemoglobin	10.6 g/dL	11.9-14.7 g/dL
Hematocrit	32.80%	37-45%
Alkaline phosphatase	234 IU/L	35-136 IU/L
Cancer antigen 125	224 U/mL	0-35 U/mL
Cancer antigen 19-9	578.83 U/mL	0-37 U/mL
Carcinoembryogenic antigen	1.74 ng/mL	<5 ng/mL
Cancer antigen 15-3	15.4 U/mL	0-38 U/mL

The levels of alpha-fetoprotein and beta-human chorionic gonadotropin were within the normal limits. Colonoscopy and gastroscopy results were normal, and the results for *Helicobacter pylori *infection were negative.

**Figure 3:**
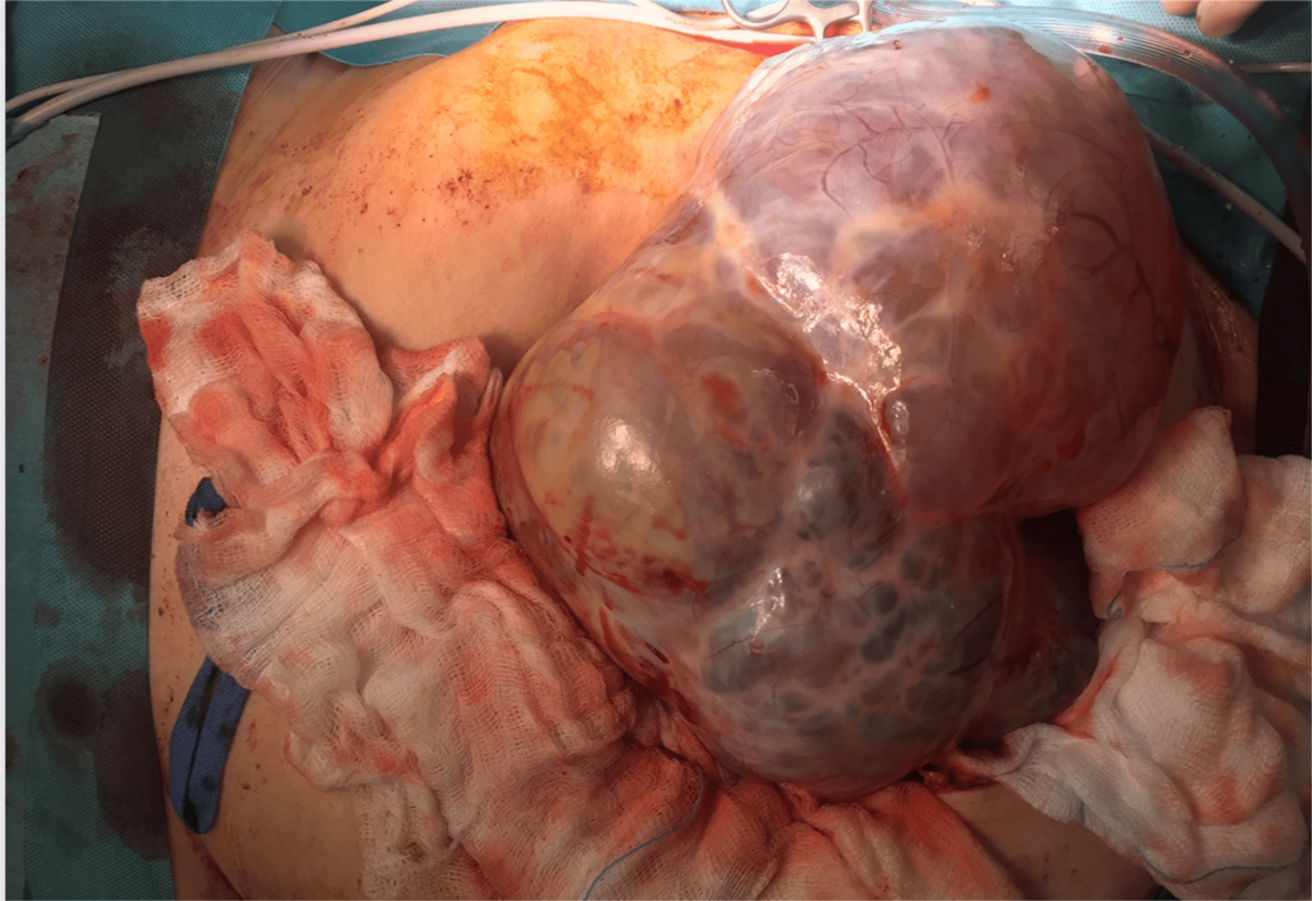
Large borderline ovarian tumor during surgery.

Based on these findings, an ovarian mass was suspected. When the patient had an exploratory laparotomy, a huge cystic tumor larger than 25 cm was discovered and removed en bloc (Figure 3).

**Figure 4:**
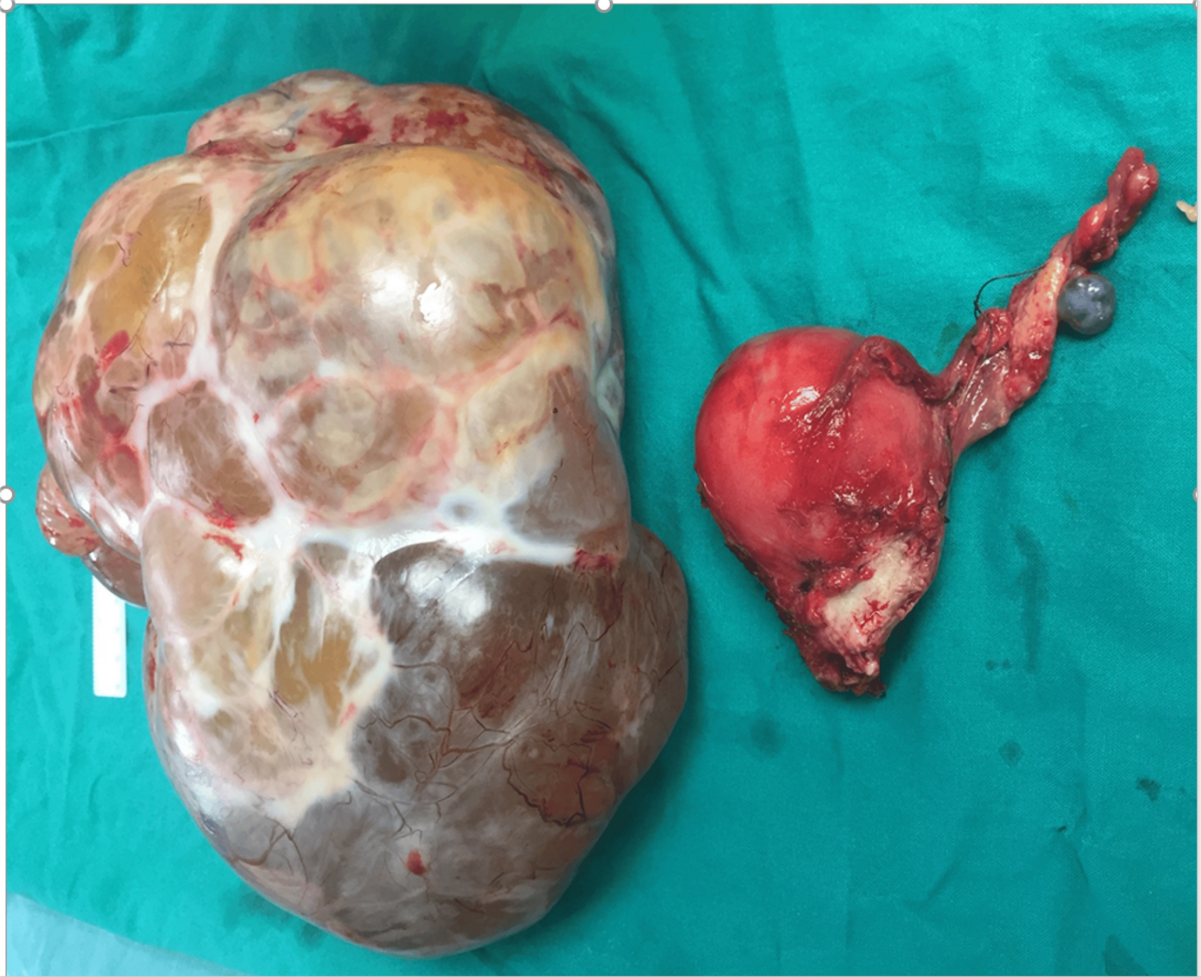
Large borderline ovarian tumor after resection along with the appendix.

Total hysterectomy with bilateral salpingo-oophorectomy and appendectomy was also performed. No other masses or mucin deposits were noticed in the peritoneal cavity, and no particular evidence of tumor metastasis in the mesentery, intestines, larger omentum, or peritoneum was found. No blood transfusion was required, and there were no difficulties intra- or post-operatively (Figure 4).

The histopathological examination revealed a large cystic mass measuring 25 x 18 x 16 cm and weighing 4 kg. The external surface of the tumor was whitish grey, smooth, and intact. The tumor displayed a multicystic surface upon dissection, with thick septa separating the cystic areas of varying sizes and viscous mucus filling them. The histological study showed a mucinous neoplasm of borderline malignancy. No signs of infiltrative growth of the tumor, intraepithelial carcinoma, or stromal microinvasion were observed.

The treatment modalities were curative, and no further management was required. The postoperative period was uneventful, and the patient was discharged on the sixth day after surgery. At the one-month postoperative follow-up, all medical examinations were within normal ranges, including hematocrit and tumor marker levels. No recurrence has occurred 1.5 years after the operation.

## Discussion

Ovarian mucinous borderline tumours are uncommon epithelial tumours that lie between invasive mucinous carcinomas and benign mucinous cystadenomas and usually appear as large cystic masses containing gelatinous fluid. According to the epidemiologic data, borderline ovarian tumors are most commonly detected in women of reproductive age, usually in the third to fifth decade of life, and account for roughly 10-15% of all borderline ovarian tumours [4,5]. Most of these tumours are of low stage at the time of diagnosis, and hence, timely and proper treatment ensures a good prognosis and a low recurrence rate [1-3]. Our case report confirms the rarity and peculiarity of this presentation. The patient was a 61-year-old postmenopausal woman who remained asymptomatic until the tumour was incidentally found, despite its large size. At this age, a malignant ovarian epithelial tumour would more commonly be suspected. Two more cases of borderline ovarian tumors in women of advanced age have been reported in the literature, one was 69 years old [6] and the other was 93 years old [7]. Borderline ovarian tumors have low malignancy potential and occasionally have intraperitoneal spread. This group of neoplasms exhibits behaviour that is intermediate between benign cystadenomas and invasive carcinomas [1-3]. The vast majority are histologically serous or mucinous tumours, with a small minority of seromucinous, endometrioid, clear cell, and transitional (Brenner) tumours. There are two types of mucinous borderline ovarian tumors: endocervical-like type (15%) and gastrointestinal (or intestinal) type (85%), depending on the histological architecture and type of tumour cells [1-3]. Borderline ovarian tumors typically range in size from 10 to 30 cm and are unilateral [8]. Before being diagnosed, about 30% of patients with borderline ovarian tumors have no symptoms, and approximately 50-60% of the patients present with complaints of abdominal distention and/or pelvic pain along with normal or slightly increased cancer antigen-125 levels [1-3]. Clinical symptoms include vaginal bleeding, palpation of a mass, and distension and pain in the abdomen [1,2]. However, the patient in this case remained asymptomatic before the tumour was found incidentally, despite the large size of the tumour that had occupied the entire abdomen. For big lesions, the absence of symptoms is rare and indicates a progressive expansion. A large mass generally compresses adjacent organs, and especially a pelvic mass may result in bladder displacement with polyuria, stomach compression with early satiety, calyceal-pelvic dilatation, and colonic compression with constipation.

There are no specific radiological features selectively describing borderline ovarian tumors, although multilocular cystic structures can be seen on CT-scan and ultrasound images; it is still difficult to differentiate borderline ovarian tumors from mucinous carcinomas, which makes the diagnostic process more difficult [9]. Despite the fact that a definite preoperative diagnosis is difficult, a hypothetical diagnosis can be made according to clinical manifestations, tumour markers, and imaging findings. Transvaginal ultrasound, computed tomography, and magnetic resonance imaging are crucial methods to assist in the presumptive diagnosis of such tumours. Nonetheless, a pathology diagnosis ought to bolster the final diagnosis, including the existence of microinvasion. The cornerstone treatment for borderline ovarian tumors is surgery, with careful staging being necessary to rule out invasion or metastasis [10]. The tumor's features, the patient's age, and her wish to maintain her fertility are taken into account when selecting the treatment plan. There are two possible types of operation. The first is a conservative procedure that preserves at least a portion of the uterus and the unilateral ovary, sometimes referred to as fertility-preserving surgery. Younger patients with endocrine or reproductive needs can benefit from it, particularly if the tumor is confined to the ovary [10]. The second procedure is more appropriate for patients who no longer want children because it is a radical one that involves at least a hysterectomy and bilateral adnexectomy. As many patients are still of reproductive age, there is considerable interest in conservative management with preservation of the childbearing ability. In case of the mucinous type of borderline ovarian tumor, the appendix might also be removed to exclude the possibility of ovarian metastasis of mucinous tumours of the appendix. To date, there is no proven benefit from any adjuvant therapy (chemotherapy, radiotherapy) in the case of borderline ovarian tumor. Chemotherapy after surgery is generally not advised because research has shown that it might cause complications and raise the death rate, but it does not always enhance the prognosis of patients [8]. Since our patient was not of reproductive age, she underwent a total hysterectomy with bilateral salpingo-oophorectomy. Moreover, this treatment approach was chosen considering that after conservative surgery, the median number of relapses is nearly 15%, compared to 5% in cases of radical surgery [7].

The prognosis for borderline tumors is favourable, and they have a low recurrence rate (5-20%). If they occur, they typically stay borderline. Stated differently, although malignant transformation is uncommon, recurrence, disease progression, and mortality remain possible outcomes [1-3]. Cancer antigen-125 levels have been reported as an independent risk factor for the recurrence of borderline ovarian tumors, although it is nonspecific; patients having raised levels of the cancer antigen-125 had a considerably higher chance of recurrence [11-13]. Despite the low recurrence rate, surveillance is recommended for a long time following primary treatment of borderline ovarian tumors, especially in instances that are conservatively handled or not fully staged. Therefore, even in cases with early-stage disease, long-term follow-up is advised.

## Conclusions

This case suggests that despite the elevated preoperative cancer antigen-125 levels and the large size of the tumour, early-stage detection and treatment of borderline ovarian tumors is crucial, as they can have a more favourable prognosis. Although this tumour type is not frequent in women of advanced age, preoperative assessment of imaging findings and serum tumour levels could help in the diagnosis. This case highlights several crucial concerns, including the following: (1) borderline ovarian tumors can grow significantly in size without producing symptoms, (2) imaging may not always distinguish borderline ovarian tumors from other entities, and (3) total surgical excision is still essential for the best results. This study adds to the expanding corpus of research highlighting the need for awareness, even in asymptomatic women, and the many clinical manifestations of borderline ovarian tumors.
